# Supplementary material for: Predictive Models of within- and between-Species SARS-CoV-2 Transmissibility
Source: Viruses. 2022 Jul 19;14(7):1565. doi: 10.3390/v14071565 (PMC9318986; doi:10.3390/v14071565)
Supplement: Supplementary file 1 [file viruses-14-01565-s001.zip › Table_S1.pdf]

Table S1 – List of candidate species susceptible to SARS-CoV-2 infection obtained using the large scale predictive model. Damas score represents the results obtained by Damas et al. (2020). Species without this score were absent in their sample.

Class	Order	Family	Species	Damas Score
Mammalia	Artiodactyla	Camelidae	*Camelus bactrianus*	Low
Mammalia	Artiodactyla	Camelidae	*Camelus dromedarius*	Low
Mammalia	Artiodactyla	Camelidae	*Vicugna pacos*	Medium
Mammalia	Artiodactyla	Bovidae	*Bos indicus x Bos taurus*	Medium
Mammalia	Artiodactyla	Bovidae	*Bos mutus*	Medium
Mammalia	Artiodactyla	Bovidae	*Bos taurus*	Medium
Mammalia	Artiodactyla	Bovidae	*Bubalus bubalis*	Medium
Mammalia	Artiodactyla	Bovidae	*Capra hircus*	Medium
Mammalia	Artiodactyla	Bovidae	*Ovis aries*	Medium
Mammalia	Artiodactyla	Suidae	*Sus scrofa*	Low
Mammalia	Carnivora	Felidae	*Acinonyx jubatus*	Medium
Mammalia	Carnivora	Felidae	*Felis catus*	Medium
Mammalia	Carnivora	Felidae	*Lynx canadensis*	Medium
Mammalia	Carnivora	Felidae	*Panthera pardus*	Medium
Mammalia	Carnivora	Felidae	*Puma concolor*	Medium
Mammalia	Carnivora	Felidae	*Panthera leo*	-
Mammalia	Carnivora	Canidae	*Canis lupus dingo*	Low
Mammalia	Carnivora	Canidae	*Vulpes vulpes*	Low
Mammalia	Carnivora	Mustelidae	*Enhydra lutris kenyoni*	Very low
Mammalia	Carnivora	Mustelidae	*Lontra canadensis*	-
Mammalia	Carnivora	Mustelidae	*Mustela erminea*	Very low
Mammalia	Carnivora	Mustelidae	*Mustela putorius furo*	Very low
Mammalia	Carnivora	Mustelidae	*Neovison vison*	Very low
Mammalia	Carnivora	Otariidae	*Callorhinus ursinus*	Very low
Mammalia	Carnivora	Otariidae	*Eumetopias jubatus*	Very low
Mammalia	Carnivora	Otariidae	*Zalophus californianus*	Very low
Mammalia	Carnivora	Phocidae	*Mirounga leonina*	-
Mammalia	Carnivora	Phocidae	*Neomonachus schauinslandi*	Very low
Mammalia	Carnivora	Odobenidae	*Odobenus rosmarus divergens*	Very low
Mammalia	Carnivora	Phocidae	*Phoca vitulina*	Very low
Mammalia	Carnivora	Ursidae	*Ursus arctos horribilis*	Low
Mammalia	Cetacea	Balaenopteridae	*Balaenoptera a. scammoni*	High
Mammalia	Cetacea	Monodontidae	*Delphinapterus leucas*	High
Mammalia	Cetacea	Delphinidae	*Globicephala melas*	High
Mammalia	Cetacea	Delphinidae	*Lagenorhynchus obliquidens*	High
Mammalia	Cetacea	Lipotidae	*Lipotes vexillifer*	High
Mammalia	Cetacea	Monodontidae	*Monodon monoceros*	High
Mammalia	Cetacea	Phocoenidae	*Neophocaena a. asiaeorientalis*	High
Mammalia	Cetacea	Delphinidae	*Orcinus orca*	High
Mammalia	Cetacea	Phocoenidae	*Phocoena sinus*	-
Mammalia	Cetacea	Physeteridae	*Physeter catodon*	Medium
Mammalia	Cetacea	Delphinidae	*Tursiops truncatus*	High
Mammalia	Chiroptera	Vespertilionidae	*Eptesicus fuscus*	Very low
Mammalia	Chiroptera	Phyllostomidae	*Phyllostomus discolor*	Very low
Mammalia	Chiroptera	Pteropodidae	*Pteropus alecto*	Low
Mammalia	Chiroptera	Pteropodidae	*Pteropus vampyrus*	Low

Mammalia	Chiroptera	Pteropodidae	*Rousettus aegyptiacus*	Low
Mammalia	Diprotodontia	Phascolarctidae	*Phascolarctos cinereus*	Very low
Mammalia	Diprotodontia	Vombatidae	*Vombatus ursinus*	Very low
Mammalia	Lagomorpha	Leporidae	*Oryctolagus cuniculus*	Medium
Mammalia	Lagomorpha	Ochotonidae	*Ochotona princeps*	Low
Mammalia	Perissodactyla	Equidae	*Equus asinus*	Low
Mammalia	Perissodactyla	Equidae	*Equus caballus*	Low
Mammalia	Perissodactyla	Equidae	*Equus przewalskii*	Low
Mammalia	Perissodactyla	Rhinocerotidae	*Ceratotherium simum simum*	Low
Mammalia	Pholidota	Manidae	*Manis javanica*	Very low
Mammalia	Primates	Aotidae	*Aotus nancymaae*	Medium
Mammalia	Primates	Cercopithecidae	*Cercocebus atys*	Very high
Mammalia	Primates	Cercopithecidae	*Chlorocebus sabaeus*	Very high
Mammalia	Primates	Hominidae	*Gorilla gorilla gorilla*	Very high
Mammalia	Primates	Hominidae	*Homo sapiens*	Very high
Mammalia	Primates	Cercopithecidae	*Macaca fascicularis*	Very high
Mammalia	Primates	Cercopithecidae	*Macaca mulatta*	Very high
Mammalia	Primates	Cercopithecidae	*Macaca nemestrina*	Very high
Mammalia	Primates	Cercopithecidae	*Mandrillus leucophaeus*	Very high
Mammalia	Primates	Cheirogaleidae	*Microcebus murinus*	Low
Mammalia	Primates	Hylobatidae	*Nomascus leucogenys*	Very high
Mammalia	Primates	Hominidae	*Pan paniscus*	Very high
Mammalia	Primates	Hominidae	*Pan troglodytes*	Very high
Mammalia	Primates	Cercopithecidae	*Papio anubis*	Very high
Mammalia	Primates	Cercopithecidae	*Piliocolobus tephrosceles*	Very high
Mammalia	Primates	Cercopithecidae	*Rhinopithecus roxellana*	Very high
Mammalia	Primates	Cebidae	*Saimiri boliviensis boliviensis*	Medium
Mammalia	Primates	Cebidae	*Sapajus apella*	Medium
Mammalia	Primates	Cercopithecidae	*Theropithecus gelada*	Very high
Mammalia	Primates	Cercopithecidae	*Trachypithecus francoisi*	-
Mammalia	Primates	Hylobatidae	*Hylobates moloch*	-
Mammalia	Primates	Cebidae	*Callithrix jacchus*	Medium
Mammalia	Primates	Hominidae	*Pongo abelii*	Very high
Mammalia	Primates	Galagidae	*Otolemur garnettii*	Very low
Mammalia	Primates	Indriidae	*Propithecus coquereli*	High
Mammalia	Proboscidea	Elephantidae	*Loxodonta africana*	Low
Mammalia	Rodentia	Cricetidae	*Peromyscus leucopus*	Low
Mammalia	Rodentia	Cricetidae	*Peromyscus maniculatus bairdii*	Medium
Mammalia	Rodentia	Cricetidae	*Cricetulus griseus*	High
Mammalia	Rodentia	Cricetidae	*Mesocricetus auratus*	Medium
Mammalia	Rodentia	Cricetidae	*Microtus ochrogaster*	Low
Mammalia	Rodentia	Muridae	*Mastomys coucha*	Very low
Mammalia	Rodentia	Muridae	*Mus caroli*	Very low
Mammalia	Rodentia	Muridae	*Mus pahari*	Very low
Mammalia	Rodentia	Muridae	*Rattus rattus*	-
Mammalia	Rodentia	Muridae	*Mus musculus*	Very low
Mammalia	Rodentia	Muridae	*Grammomys surdaster*	Very low
Mammalia	Rodentia	Spalacidae	*Nannospalax galili*	High
Mammalia	Rodentia	Sciuridae	*Marmota flaviventris*	Medium
Mammalia	Rodentia	Dipodidae	*Jaculus jaculus*	Very low
Mammalia	Rodentia	Bathyergidae	*Heterocephalus glaber*	Medium

Mammalia	Rodentia	Sciuridae	*Ictidomys tridecemlineatus*	Medium
Mammalia	Rodentia	Sciuridae	*Urocitellus parryii*	Medium
Mammalia	Rodentia	Heteromyidae	*Dipodomys ordii*	Medium
Mammalia	Scandentia	Tupaiidae	*Tupaia chinensis*	Very low
Mammalia	Sirenia	Trichechidae	*Trichechus manatus latirostris*	Low
Aves	Passeriformes	Thraupidae	*Camarhynchus parvulus*	Very low
Aves	Passeriformes	Paridae	*Cyanistes caeruleus*	Very low
Aves	Passeriformes	Muscicapidae	*Ficedula albicollis*	Very low
Aves	Passeriformes	Fringillidae	*Serinus canaria*	Very low
Aves	Passeriformes	Sturnidae	*Sturnus vulgaris*	Very low
Aves	Passeriformes	Passerellidae	*Zonotrichia albicollis*	Very low
Reptilia	Squamata	Lacertidae	*Zootoca vivipara*	Very low
