# Supplementary figures and images for: Predictive Models of within- and between-Species SARS-CoV-2 Transmissibility
Source: Viruses. 2022 Jul 19;14(7):1565. doi: 10.3390/v14071565 (PMC9318986; doi:10.3390/v14071565)

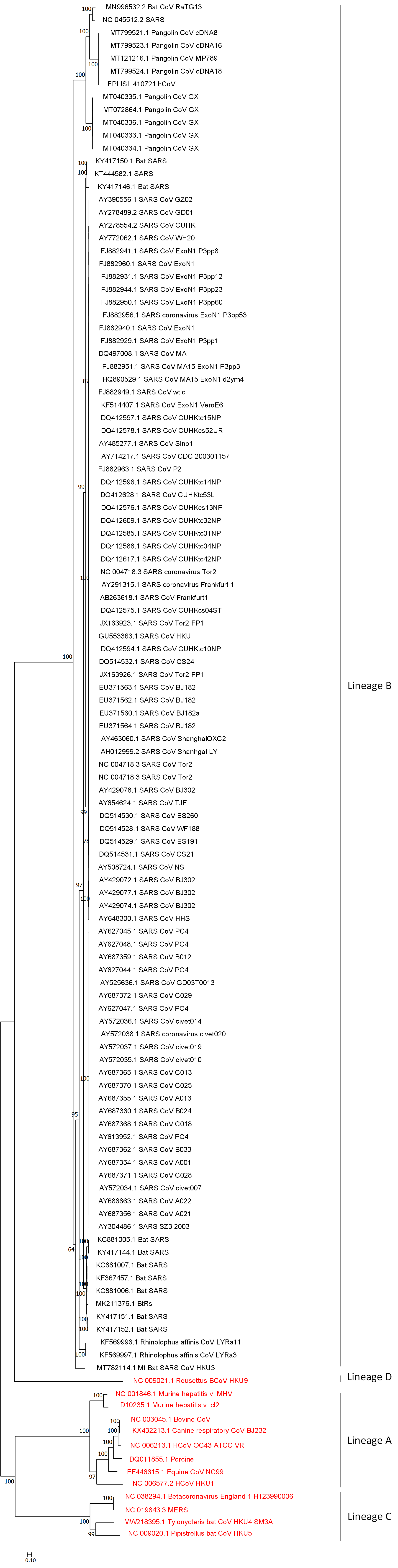

Supplement: Supplementary file 1 [file viruses-14-01565-s001.zip › Figure_S1.tif]

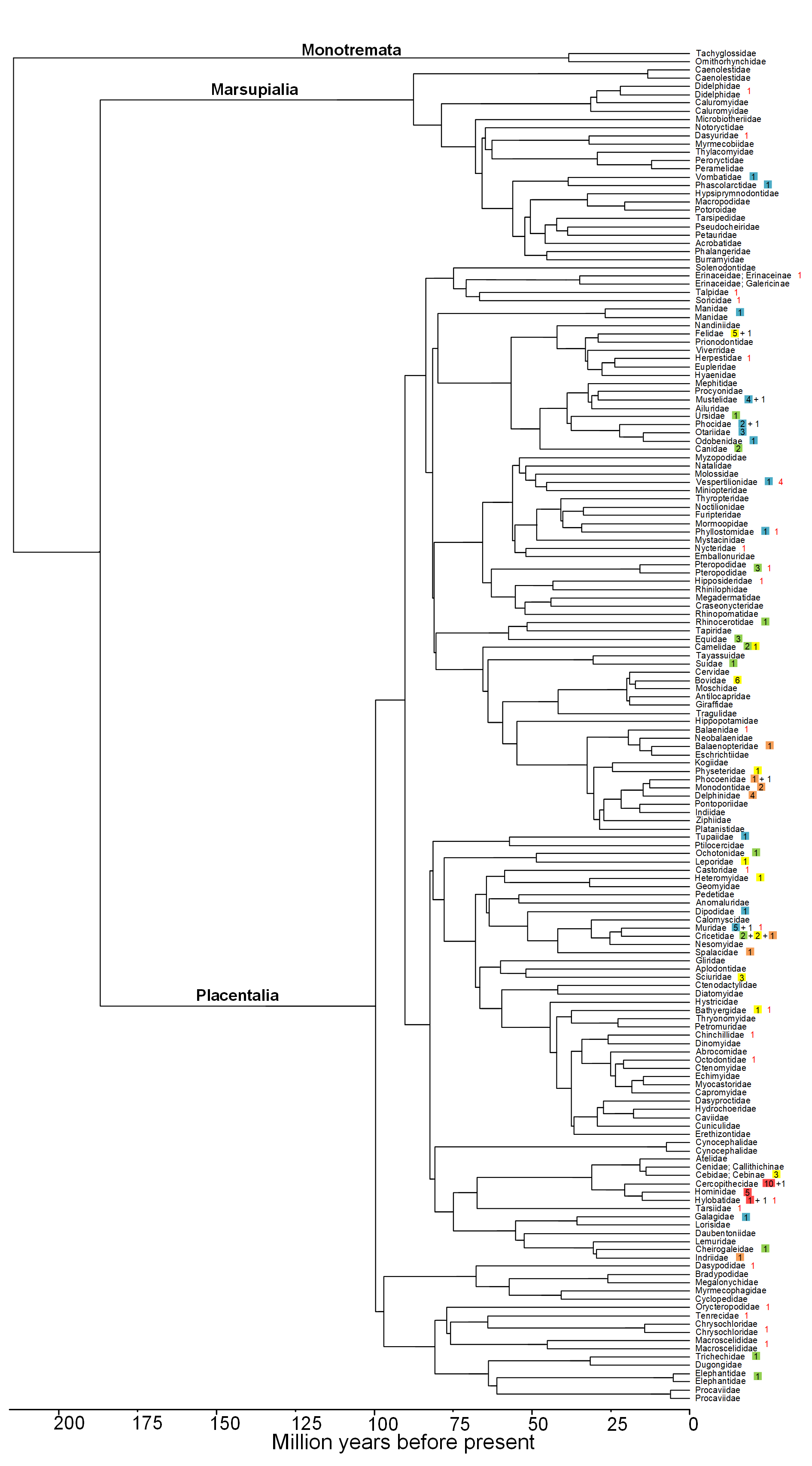

Supplement: Supplementary file 1 [file viruses-14-01565-s001.zip › Figure_S2.tif]

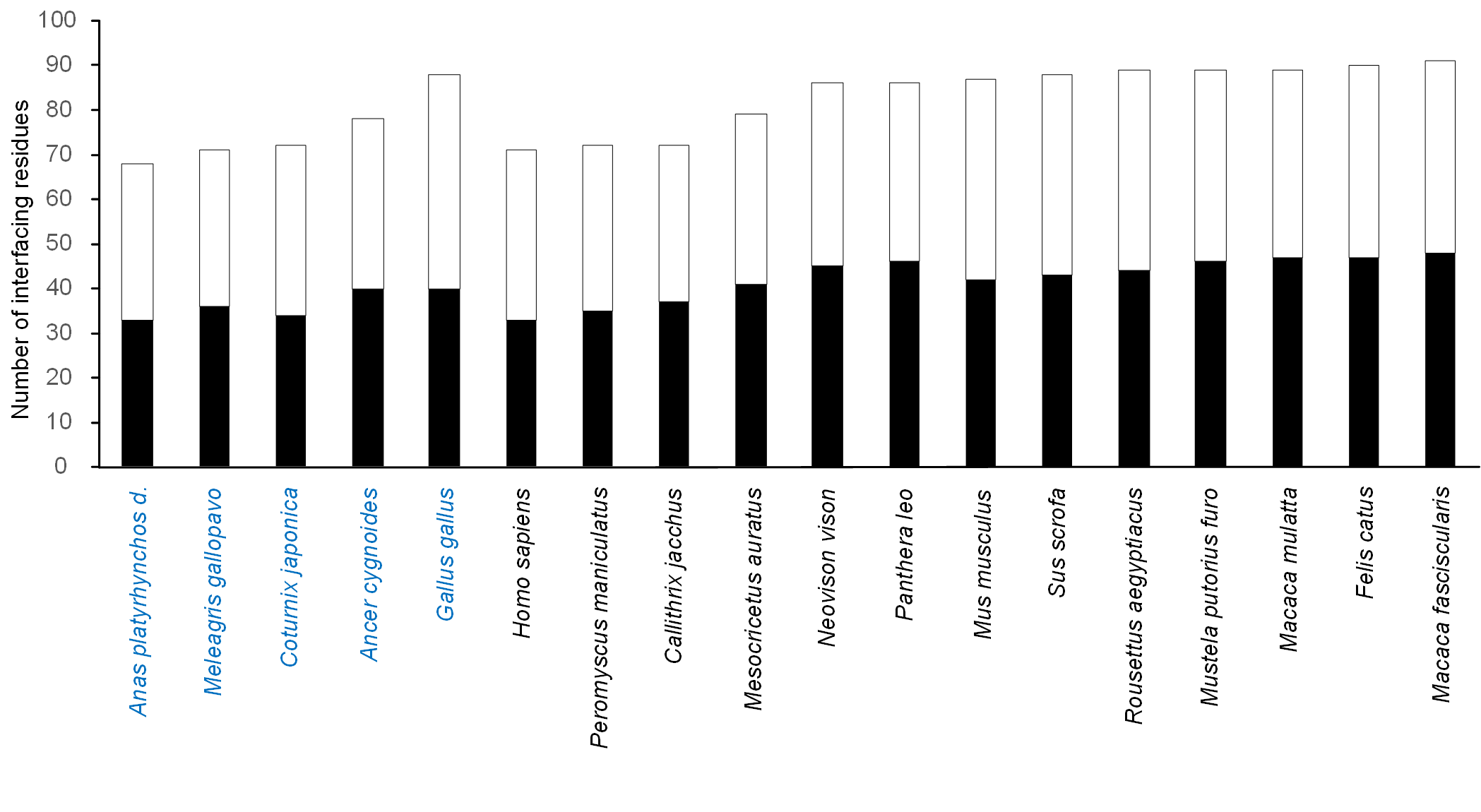

Supplement: Supplementary file 1 [file viruses-14-01565-s001.zip › Figure_S3.tif]
